# Increased seroprevalence of IgG-class antibodies against cytomegalovirus, parvovirus B19, and varicella-zoster virus in women working in child day care

**DOI:** 10.1186/1471-2458-12-475

**Published:** 2012-06-22

**Authors:** Gini G C van Rijckevorsel, Lian P M J Bovée, Marjolein Damen, Gerard J B Sonder, Maarten F Schim van der Loeff, Anneke van den Hoek

**Affiliations:** 1 Public Health Service Amsterdam, Department of Infectious Diseases, Nieuwe Achtergracht 100, Amsterdam, WT, 1018, The Netherlands; 2 Public Health Laboratory, Cluster of Infectious Diseases, Public Health Service of Amsterdam, Nieuwe Achtergracht 100, Amsterdam, WT, 1018, The Netherlands; 3 Department of Internal Medicine, Division of Infectious Diseases, Tropical Medicine and AIDS, Academic Medical Centre, Meibergdreef 9, Amsterdam, 1105, AZ, The Netherlands; 4 Public Health Service Amsterdam, Cluster Infectious Diseases, Department of Research, Nieuwe Achtergracht 100, Amsterdam, WT, 1018, The Netherlands; 5 Public Health Service Amsterdam, Nieuwe Achtergracht 100, Amsterdam, WT, 1018, the Netherlands

**Keywords:** Cytomegalovirus, Parvovirus B19, Varicella-zoster virus, Seroprevalence, Child day care, Occupational risk

## Abstract

**Background:**

Primary maternal infection with cytomegalovirus (CMV), parvovirus B19 (B19V), and varicella-zoster virus (VZV) may result in adverse pregnancy outcomes like congenital infection or foetal loss. Women working in child day care have an increased exposure to CMV, B19V, and VZV. By comparing the seroprevalence of IgG-class antibodies against CMV, VZV and B19V in female day care workers (DCW) with the seroprevalence in women not working in day care this study aimed to assess the association between occupation and infection.

**Methods:**

A cross-sectional design was used. Out of a random sample of 266 day care centres, demographic data, data on work history, and blood samples were collected from 285 women from 38 centres. In addition, blood samples and basic demographics from women who participated in a cross-sectional survey of the Amsterdam population (2004) were used. All blood samples were tested for IgG-class antibodies against CMV, B19V, and VZV.

**Results:**

Twenty-seven percent of the DCW were still susceptible to B19V or CMV. Working in day care was independently associated with B19V infection in all DCW (prevalence ratio [PR] 1.2; 95 % CI 1.1–1.3), and with CMV infection in DCW of European origin only (PR 1.7; 95 % CI 1.3–2.3). Almost all women born outside Europe tested seropositive for CMV (96 %). All DCW tested seropositive for VZV, compared to only 94 % of the women not working in day care.

**Conclusion:**

This study confirms the clear association between employment in child day care centres and infection with CMV and B19V. Intervention policies, like screening of new employees and awareness campaigns emphasizing hygienic measures among DCW, should be implemented urgently to improve the maternal health of these women and the health of their offspring.

## Background

Primary maternal infection with cytomegalovirus (CMV), parvovirus B19 (B19V), and varicella-zoster virus (VZV) may result in adverse pregnancy outcomes like congenital infection or foetal loss [1-4]. CMV is the most common congenital infection, occurring in 0.3 % to 1.0 % of all live births worldwide. It may lead to permanent disabilities in the unborn child, such as deafness, blindness and mental impairments [5]. Primary CMV infection results in life-long latent infection, and although congenital infection after reactivation and re-infection with a different CMV strain may occur, the risk of congenital infection is highest for seronegative women [6,7]. It is difficult to identify an acute CMV infection because the disease is asymptomatic in 90 % of individuals and the clinical signs, if present, are non-specific. In contrast, primary VZV infection almost always causes signs of disease, specifically, a generalized pruritic vesicular rash called chickenpox [3]. Primary maternal infection with VZV during the first 20 weeks of pregnancy only, can lead to foetal varicella syndrome in 1 % to 2 % of the patients, but perinatal infection can also be harmful to the newborn [3,4,8]. Like VZV, the risk of congenital B19V infection depends on the gestational age, with the greatest risk following maternal infection in the first 20 weeks. In about 5–10 % of pregnant women with primary B19V infection, transplacental transmission may lead to adverse pregnancy outcome like hydrops foetalis or foetal death [3,9]. After primary VZV or B19V infection, immunity is regarded as life-long [3,9].

In the Netherlands, most people experience primary CMV, B19V, and VZV infection during their childhood. Vaccination against VZV is not part of the national childhood vaccination program. The identification of risk groups susceptible to these infections is important to assess the risk of infection in pregnant women. The risk of exposure is generally present as CMV, B19V, and VZV circulate widely in the population, but is particularly high in child day care centres and schools. Children attending day care have significantly higher rates of CMV excretion in their saliva and urine, compared to children not attending day care [10,11]. Outbreaks of VZV and B19V occur regularly in day care centres and schools. Employees of day care centres, most of whom are women of childbearing age, are repeatedly exposed to CMV, B19V, and VZV and, therefore, are considered at risk [10,12-14]. The purpose of this study was to provide current estimates of the seroprevalence of IgG-class antibodies against CMV, VZV, and B19V in women of childbearing age working in child day care centres in Amsterdam (DCW). By comparing these seroprevalence rates with data from Amsterdam women not working in day care, we aimed to assess the association between occupation and infection in DCW. Additionally, other likely determinants of seropositivity for CMV, B19V, and VZV, such as age and ethnic origin, were investigated.

## Methods

### Study population and sampling procedure

#### *Child day care workers*

Data on child day care personnel were obtained through a cross-sectional survey carried out in 2007 by the Public Health Service of Amsterdam (GGD Amsterdam). Out of 266 day care centres on the Amsterdam municipal register, a random sample of 63 was drawn. All 63 centres were invited to participate, with a response rate of 60 %. Thirty-eight centres were then visited; all female employees present were invited to participate, and nearly all agreed, yielding a total of 285 participants. After giving informed consent, the participants were questioned via a standardized face-to-face interview concerning socio-demographics, family size, work history, and a blood sample was collected. The samples were centrifuged and frozen at −80 °C within 48 h. The following variables were considered pertinent to the study: sex, age, country of birth of the participant and her parents, when applicable the age at the time of immigration, the number and age of children living with the participant currently and/or in the past, and the number of years working as a child care worker. In the analysis only women of childbearing age (16 to 44 years) were included (n = 242).

#### *Women not working in child day care*

Data on women not working in day care came from a cross-sectional survey of the adult Amsterdam general population, the Amsterdam Health Monitor (AHM), carried out by the Public Health Service of Amsterdam in 2004 from which a serum repository was established. AHM data was collected using frequency stratification by ethnic group, and women of Turkish and Moroccan origin were oversampled. Seroprevalence reported for this group in our study did not account for the sampling method of the AHM and basic demographic data and blood samples from all female participants in that survey aged 18 to 44 years (n = 298) were included. It should not be considered as representative of women in the Amsterdam general population, but rather as representative of women not working in child day care. More information on the AHM and its serum repository is described elsewhere [15-17]. Approval for both studies was obtained from the Medical Ethics Committee of the Academic Medical Centre.

### Serological assays

All serum samples were tested for IgG-class antibodies against B19V by means of quantitative enzyme immunoassays (NovaLisa™ Parvovirus B19 recombinant IgG-ELISA; Novatec Immundiagnostica Gmbh.; Dietzenbach, Germany). In calculating seroprevalence, samples with a positive result (cut-off ≥ 9 NovaTec Units) were considered immune.

IgG-class antibodies against CMV were determined by a quantitative enzyme immunoassay (SERION ELISA *classic* Cytomegalovirus IgG/IgM; Institut Virion/Serion, Würzburg, Germany). Equivocal test results were considered seropositive.

Serum samples obtained from DCW were tested for IgG-class antibodies against VZV with quantitative enzyme immunoassays (SERION ELISA *classic* Varicella-Zoster-Virus IgG/IgM/IgA; Institut Virion/Serion, Würzburg, Germany). To estimate VZV seropositivity in women in the Amsterdam repository, plasma samples were used and tested for IgG-class antibodies against VZV with a microplate enzyme-linked immunosorbent assay system (EUROIMMUN Anti-Varicella-Zoster-Virus IgG-ELISA; Medizinische Labordiagnostika AG, Lübeck, Germany). Only women whose samples had a positive result (cut-off ≥ 110 mIU/ml) were considered immune. All assays were performed in the Public Health Laboratory in Amsterdam according to the instructions of the manufacturer.

### Statistical analysis

Prevalences (P) were compared using the chi-square test; values of p < 0.05 were considered significant. Univariable and multivariable binominal regression analysis, adjusted for day-care centre clustering, was used to estimate prevalence ratios (PR) with 95 % confidence intervals (CI) based on robust standard errors. If significant at univariable level (p < 0.1), covariates were included in a multivariable model. In the analysis the AHM data were not corrected for the sampling method. All analyses were performed in Intercooled Stata 11.1 for Windows (Stata Corp., College Station, Texas, USA).

## Results

### Characteristics of the study sample

Table 1 shows the characteristics of the total of 540 women aged 16 to 44 years included in the analyses; 55 % (298) were women from the AHM, and 45 % (242) were DCW. The median age was 32 years (interquartile range [IQR] 25–39 years). The DCW were younger than the women from the AHM (median age 29 years; IQR 24–35 years versus 35 years; IQR 28–40 years; rank sum test, p < 0.001). Most women (294; 54 %) were born in the Netherlands. Of the women born elsewhere, 37 % originated from Turkey, 27 % from Morocco, 15 % from Suriname or the Dutch Antilles, 10 % from within Europe and 11 % from other countries (Africa, Asia, Central and South America). Most DCW were born in Europe (77 %), whereas women from the Amsterdam Health Monitoring Survey were more often born outside Europe (46 %). Most immigrants (59 %) arrived in the Netherlands when they were adults; the median age at migration was 20 years (range 1–42 years). Sixty-two per cent of the women were mothers, 19 % had 1 child, 21 % had 2 children, and 14 % had 3 or more. The two groups differed in that 84 % of the women of the AHM had children compared to only 42 % of the DCW. Also, 84 % of all women born outside Europe had one or more children compared to only 47 % of all women born in the Netherlands or Europe (p < 0.001).

**Table 1:** Characteristics of 242 female child day care workers in Amsterdam, The Netherlands, compared with 298 women of the Amsterdam population (2004)

**Characteristics**	**N**	**%**	**Child Day Care Workers**		**Population sample**		
			n	%	n	%	*p* value
**Total**	540		242	44.8%	298	55.2%	
**Age**							
Median age in years	32 (IQR 25–39)	29 (IQR 24–35)	35 (IQR 28–40)	< 0.001			
**Age (in years)**							< 0.001
<20	23	4.3 %	11	4.6 %	12	4.0 %	
20–24	92	17.0 %	51	21.1 %	39	13.1 %	
25–29	107	19.8 %	69	28.5 %	40	13.4 %	
30–34	90	16.7 %	48	19.8 %	42	14.1 %	
35–39	108	20.0 %	30	12.4 %	78	26.2 %	
40–44	120	22.2 %	33	13.6 %	87	29.2 %	
**Country of birth**							< 0.001
The Netherlands	294	54.4 %	176	72.7 %	118	39.6 %	
Other European countries	25	4.6 %	11	4.6 %	14	4.7 %	
Suriname and Dutch Antilles	37	6.9 %	26	10.7 %	11	3.7 %	
Turkey	90	16.7 %	5	2.1 %	85	28.5 %	
Morocco	66	12.2 %	15	6.2 %	51	17.1 %	
Other countries	28	5.2 %	9	3.7 %	19	6.4 %	
**Country of birth by continent**							< 0.001
The Netherlands & Other European countries	319	59.1 %	187	77.3 %	132	44.3 %	
Non-European countries*	221	40.9 %	55	22.7 %	166	55.7 %	
**Age at immigration**							< 0.001
0–17 years	97	40.9 %	27	44.3 %	70	39.6 %	
18 and older	140	59.1 %	34	55.7 %	107	60.5 %	
Data missing	9	–	5	–	4	–	
Not applicable (born in the Netherlands)	294	–	176	–	118	–	
**Having children**							< 0.001
Yes	293	62.2 %	101	41.7 %	192	84.2 %	
No	177	37.8 %	141	58.3 %	36	15.8 %	
Data missing	70	–	0	–	70	–	
**Number of children**							< 0.001
0	177	32.8 %	141	58.3 %	36	12.1 %	
1	104	19.3 %	46	19.0 %	58	19.5 %	
2	113	20.9 %	35	14.5 %	78	26.2 %	
3	76	14.1 %	20	8.3 %	56	18.8 %	
Data missing	70	13.0 %	0	0 %	70	23.5 %	

* Africa, Asia and Central and South America.

IQR; Interquartile range.

### Seroprevalence of CMV IgG

No valid test results for CMV were available for 11 women (1 DCW and 10 from the general Amsterdam population survey). The seroprevalence of CMV IgG antibodies among the 529 women with valid test results was 73.0 % (95 % CI 69.0–76.7 %), and was similar among DCW and among the women not working in day care. Table 2 shows the seroprevalence by demographic characteristics and the PR from univariable analysis. Seroprevalence was significantly higher among immigrants born outside Europe (96 %) compared to women born in European countries, including the Netherlands (57 %). Nearly all of the women born in Turkey (99 %), in Morocco (98 %), or in Suriname or the Dutch Antilles (97 %) tested IgG-seropositive for CMV. Because the seroprevalence of CMV in immigrants born outside Europe approached 100 %, a subgroup analysis limited to the strata of women born in Europe was performed in a separate multivariable binominal regression model. In this model, after adjusting for working in child care, for age (in categories), and for having children, CMV seroprevalence in DCW was significantly higher than among women not working in day care centres (PR 1.7; 95 % CI 1.3–2.3; p < 0.001), and also among those having one or more children of their own (PR 1.2; 95 % CI 1.1–1.4; p = 0.03). (Data not shown).

**Table 2:** Prevalence of IgG antibodies against cytomegalovirus (CMV) by demographic characteristics in female child day care workers (2007) in Amsterdam, The Netherlands, and in women of the Amsterdam population (2004) *

Characteristics	Study Sample	CMV IgG Positive			Univariable PR (95 % CI)	
	N	n	%	*p* value		*p* value
**Total**	529	386	73.0 %			
**Working in child day care**				0.8		
No	288	209	72.6 %		1	
Yes	241	177	73.4 %		1.0 (0.9–1.1)	0.8
**Age (in years)**				0.08		
16–24	110	80	72.3 %		1	
25–34	199	135	67.8 %		0.9 (0.8–1.1)	0.4
35–44	220	171	77.7 %		1.1 (0.9–1.2)	0.3
**Country of birth**				< 0.001		
The Netherlands & Other European countries	313	178	56.9 %		1	
Non-European countries**	216	208	96.3 %		0.6 (0.5–0.7)	< 0.001
**Age at immigration**				<0.001		
0–17 years	95	88	92.6 %		1	
18 and older	138	129	93.5 %		1.0 (0.9–1.1)	0.8
Data Missing	8	8	100 %		–	
Not applicable (born in the Netherlands)	288	161	55.9 %		–	
**Having children**				< 0.001		
No	175	113	64.6 %		1	
Yes	288	236	81.9 %		1.3 (1.1–1.4)	< 0.001
Data missing	66	39	56.1 %		–	
**Number of children**				< 0.001		
0	175	113	64.6 %		1	
1	103	76	73.8 %		1.1 (1.0–1.3)	0.1
2	111	90	81.8 %		1.3 (1.1–1.5)	0.003
3	74	70	94.6 %		1.5 (1.3–1.7)	< 0.001
Number of children missing	66	37	56.1 %		–	

* All analysis adjusted for day-care centre clustering.

** Africa, Asia and Central and South America.

### Seroprevalence of B19V IgG

The seroprevalence of B19V IgG antibodies among all 540 women was 65.9 % (95 % CI 61.8–69.9 %). The seroprevalence among DCW (73 %) was significantly higher compared to the women not working in day care (60 %; PR 1.2, 95 % CI 1.1–1.4; p = 0.003). Table 3 shows the seroprevalence per variable, as well as the results of the univariable and multivariable analyses. In a multivariable model, working at a child care centre was an independent determinant of B19V IgG-seropositivity (PR 1.2, 95 % CI 1.1–1.3; p = 0.002). Also, in this model, being a parent of one or more children was significantly associated with B19V IgG-seropositivity (PR 1.2; 95 % CI 1.0–1.3; p = 0.02). No other independent risk factors were identified.

**Table 3:** Prevalence of IgG antibodies against parvovirus B19 by demographic characteristics in female day care workers (2007) in Amsterdam, The Netherlands, and in women of the Amsterdam population (2004)*

**Characteristics**	**Study Sample**	**Parvovirus B19 IgG Positive**			**Univariable PR (95 % CI)**		**Multivariable PR (95% CI**	
	N	n	%	*p* value		*p* value		*p* value
**Total**	540	356	65.9 %					
**Working in child day care**				0.003				
No	298	180	60.4 %		1		1	
Yes	242	176	72.7 %		1.2 (1.1–1.4)	0.002	1.2 (1.1–1.4)	0.002
**Age (in years)**				0.18				
16–24	113	72	63.7 %		1		1	
25–34	199	141	70.9 %		1.1 (1.0–1.3)	0.2	1.1 (0.9–1.3)	0.2
35–44	228	143	62.7 %		1.0 (0.8–1.2)	0.9	1.0 (0.8–1.3)	0.7
**Country of birth**				0.75				
The Netherlands & Other European countries	319	212	66.5 %		1		1	
Non-European countries**	221	144	65.2 %		1.0 (0.9–1.1)	0.73	1.1 (0.9–1.5)	0.08
**Age at immigration**				0.12				
0–17 years	97	71	73.2 %		1			
18 and older	141	88	62.4 %		0.9 (0.7–1.0)	0.07		
Data missing	8	3	37.5 %		–			
Not applicable (born in the Netherlands)	294	194	66.0 %		–			
**Having children**				0.09				
No	177	114	64.1 %		1		1	
Yes	293	203	69.3 %		1.1 (0.9–1.2)	0.27	1.2 (1.0–1.3)	0.02
Data missing	70	39	55.7 %		–		–	
**Number of children**				0.24			–	
0	177	114	64.4 %		1			
1	104	73	70.2 %		1.1 (0.9–1.3)	0.26		
2	113	80	70.8 %		1.1 (0.9–1.3)	0.28		
3	76	50	65.8 %		1.0 (0.8–1.2)	0.82		
Number of children missing	70	39	55.7 %		–			

* All analysis adjusted for day-care centre clustering.

** Africa, Asia and Central and South America.

### Seroprevalence of VZV antibodies IgG

All 540 women were tested for IgG antibodies against VZV. The seroprevalence of VZV IgG antibodies in the study sample was 96.5 % (95 % CI 94.6–97.7 %). All 242 DCW were seropositive for VZV IgG antibodies (95 % CI 98.7–100.0 %), and although women from the Amsterdam general population had a high VZV IgG seroprevalence (93.6 %; 95 % CI 90.2–96.2 %), this was significantly lower than among the DCW (p < 0.001). In view of the 100 % VZV seropositivity among DCW, no overall logistic regression analysis was performed. In a multivariable model restricted to the women of Amsterdam Health Monitoring Survey (no independent predictors for seropositivity were found. (Data not shown).

## Discussion

This study demonstrates obvious differences in CMV, B19V and VZV seroprevalence between women working in Amsterdam day care centres and those who are not. In the Netherlands a population-based percentage of CMV seroprevalence is not available, yet the overall CMV seroprevalence found in this study (73 %) corresponds to previous estimates in pregnant women in the Amsterdam area [18]. In our study, CMV seroprevalence was strongly related to ethnic background; among non-European women CMV seroprevalence was much higher (96 %) than among European women (57 %) and this difference was constant across all age groups. It is well known that CMV seroprevalence varies worldwide, and is related to geographic, ethnic and social factors [19,20]. As a consequence of the very high CMV seroprevalence among non-European women, working in day care appeared not to be related to CMV seropositivity in this group. However, within the group of women of European origin, CMV seroprevalence differed considerably between those working in child care (68 %; 95 % CI 61–74 %) and those who were not (42 %; 95 % CI 32–50 %), and working in child care was independently associated with CMV IgG-seropositivity (PR 1.7) among European DCW. Whilst the same association was not found for non-European DCW born (because of their high background seropositivity), they surely have a similar occupational risk of (re-)infection to that of their European colleagues.

Unlike CMV, in this study B19V seropositivity did not depend on ethnic background, although worldwide geographic differences in B19V seroprevalence (with lower B19V seroprevalence in tropical regions) are described [20]. The B19V seroprevalence in all women of childbearing age was 66 %, in line with previous estimates in the overall Amsterdam population (61 %; 95 % CI 57–64 %) [16]. However, DCW had a significantly higher seroprevalence (73 %) compared to women not working in day care (60 %). Apart from working with children (PR 1.2), being a parent of one or more children was also associated with B19V seropositivity (PR1.2).

In this study an association between working in day care and VZV seroprevalence was not shown. Although VZV seroprevalence differed significantly between DCW (100 %) and women not working in child care (94 %) it was not possible to control for likely confounders such as age or ethnic background. Whereas VZV seroprevalence in Dutch adults is nearly 95–100 %, which is typical for adults born in a temperate climate, VZV seroprevalence in immigrants from (sub)tropical countries is often lower [21]. Remarkably, in this study all DCW, including immigrant DCW, tested positive for VZV. Although it seems plausible that some susceptible DCW may have contracted VZV after they started working in child care, data on the incidence of chickenpox in this group were not available, nor were data on the VZV serostatus at the start of the women’s employment in child care. It is likely that a boosting effect from the occupational exposure to children infected by VZV has also contributed to the 100 % seropositivity found among DCW [22,23]. Lastly, although the manufacturers of the two different enzyme immunoassays (VIRION and EUROIMMUN) used for the two serum samples groups quote similarly high sensitivity (>94 %), a discrepancy between the two tests may have affected the outcome.

A limitation in this study is that two demographically different populations were studied. Although the multivariable regression models adjusted for some important confounders like age, country of birth and having children, it is possible that other confounders, like socio-economic factors were missed. Also the sampling data of the populations differed (2004 and 2008), however the effect of this difference is likely to be negligible.

Despite these limitations, our results confirm that working in day care is independently associated with CMV and B19V infection. Although the occupational risk of infection in child care is not new and has been described since the 1990’s [10,13,14,24-29], this knowledge has not contributed to the implementation of effective preventive policies for this particular risk group. For VZV, a safe and effective vaccine is available and although some countries have adopted guidelines to screen and vaccinate risk groups (like healthcare workers) this is not applicable to DCW [30,31]. As a consequence, pregnant DCW exposed to chickenpox still need very rapid testing, and seronegative women require post-exposure prophylaxis with human varicella-human immunoglobulin within 72 h. For CMV and B19V, vaccines are not available, although the development of a vaccine against CMV is in progress [27,32]. Female DCW should be considered a risk group eligible for vaccination once the vaccine becomes available. Until that time, other preventive strategies are necessary, such as awareness campaigns to ensure pregnant women are alerted to the risks associated with exposure to CMV or B19V. This is important as several studies have described a lack of knowledge, not only among risk groups, but also among physicians about the effects of these infections during pregnancy [33-35]. Screening should be considered, especially in those who are pregnant or are trying to become pregnant, as knowledge of one’s serostatus might enhance the effect of behavioural interventions and adherence to hygiene measures such as hand washing after diaper changing. In addition, the employer, the occupational physician, and the pregnant employee who is susceptible to CMV or B19V infection could agree on alternative work during at least part of the pregnancy.

## Conclusion

This study shows clearly the association between employment in child day care centres and CMV and B19V infection. Also a considerable number of female DCW of childbearing age remain susceptible to B19V infection. To reduce the risk of congenital infection, widespread implementation of intervention policies, like screening of new employees (and if applicable, VZV vaccination), behavioural interventions and awareness campaigns among DCW, are strongly recommended to improve the maternal health of these women and the health of their offspring.

## Competing interests

The authors declare that they have no competing financial or other interests.

## Authors’ contributions

LB and GGCvR performed the data collection. GGCvR performed the data analysis and wrote the first draft of the manuscript. MD advised and supervised the carrying out of the immunoassays. MSvdL contributed to the statistical analysis. GBS and AvdH made substantial changes to the manuscript. All authors read and approved the final manuscript.

## Pre-publication history

The pre-publication history for this paper can be accessed here:

http://www.biomedcentral.com/1471-2458/12/475/prepub
